# Joint modeling of correlated binary outcomes: The case of contraceptive use and HIV knowledge in Bangladesh

**DOI:** 10.1371/journal.pone.0190917

**Published:** 2018-01-19

**Authors:** Di Fang, Renyuan Sun, Jeffrey R. Wilson

**Affiliations:** 1 Department of Agricultural Economics and Agribusiness, University of Arkansas, Fayetteville, AR, United States of America; 2 School of Mathematical and Statistical Science, Arizona State University, Tempe, AZ, United States of America; 3 Department of Economics, Arizona State University, Tempe, AZ, United States of America; Public Library of Science, UNITED KINGDOM

## Abstract

Recent advances in statistical methods enable the study of correlation among outcomes through joint modeling, thereby addressing spillover effects. By joint modeling, we refer to simultaneously analyzing two or more different response variables emanating from the same individual. Using the 2011 Bangladesh Demographic and Health Survey, we jointly address spillover effects between contraceptive use (CUC) and knowledge of HIV and other sexually transmitted diseases. Jointly modeling these two outcomes is appropriate because certain types of contraceptive use contribute to the prevention of HIV and STDs and the knowledge and awareness of HIV and STDs typically lead to protection during sexual intercourse. In particular, we compared the differences as they pertained to the interpretive advantage of modeling the spillover effects of joint modeling HIV and CUC as opposed to addressing them separately. We also identified risk factors that determine contraceptive use and knowledge of HIV and STDs among women in Bangladesh. We found that by jointly modeling the correlation between HIV knowledge and contraceptive use, the importance of education decreased. The HIV prevention program had a spillover effect on CUC: what seemed to be impacted by education can be partially contributed to one’s exposure to HIV knowledge. The joint model revealed a less significant impact of covariates as opposed to both separate models and standard models. Additionally, we found a spillover effect that would have otherwise been undiscovered if we did not jointly model. These findings further suggested that the simultaneous impact of correlated outcomes can be adequately addressed for the commonality between different responses and deflate, which is otherwise overestimated when examined separately.

## Introduction

Current Contraceptive Use (CUC) is highly encouraged by the Bangladesh government as a means of reducing fertility rates over the long term [1, 2]. Presently, approximately 61% of married women in Bangladesh currently use some form of contraceptive [1]. Notably, the birth control pill is by far the most widely used method (27%), followed by injectable (11%), condoms (6%), and female sterilization (5%) [3]. Furthermore, contraceptive use varies by age and place of residence, with higher usage in urban areas. To illustrate, the use of condoms in urban areas is 10%, versus rural areas, which is 4% [3]. Additionally, the use of condoms increases with education level and wealth quintile, while the use of injectable declines as household income increases [4].

A natural by-product of contraceptive use, particularly condom use is the reduction of human immunodeficiency virus (HIV). Accordingly, HIV prevention programs focus on two important aspects of behavior: limiting the number of sexual partners and promoting the use of condoms [3]. The awareness of HIV among ever-married women varies by age and marital status, with older women and women who are divorced, separated, or widowed less likely to know about HIV [1]. Knowledge of HIV prevention methods among both women and men is higher in urban areas over rural areas, among those with the highest level of education, and among those from the wealthiest households [3]. Even though the prevalence of HIV is low in Bangladesh (less than 1%), the country is particularly vulnerable to an HIV epidemic due to poverty, overpopulation, and gender inequality [3]. In light of this, the Bangladesh government and many other developing countries are calling for an in-depth study of both preventions of HIV and control of fertility rate [1].

Most studies ignore the interdependency between these two correlated outcomes and treat them as separate events, which has the tendency to lead to false policy recommendations due to the obvious connection between the two events [5–8]. Therefore, a systematic study of both HIV prevention and contraceptive use is crucial to account for the overlapping effects from one event to the other, while also exploring the underlying driving factors [8–11]. Thus, to conduct our study, we did two sets of models: modeling HIV knowledge and CUC separately in two hierarchical logistic regression models and jointly model both CUC and HIV knowledge. Each of these hierarchical logistic regressions addresses intra-class correlation. The motivation for a hierarchical logistic regression model is to address the random effect due to the 600 regions in Bangladesh. The joint modeling addresses the correlation of the two responses with the same set of covariates. Together, our research contributes to the practice of epidemiology by addressing a gap in the use of reliable statistical techniques that analyze the correlation of two binary outcomes at the same time, as well as the correlation resulting from similar administrative areas as the effectiveness of contraceptive use and HIV prevention is examined in Bangladesh.

The remainder of this paper is composed as follows: we first provided a review of joint modeling and concentrated on two binary outcomes, HIV and CUC. We used the 2011 Bangladesh Demographic and Health Survey data as a means to address CUC and HIV knowledge jointly. We provided a clear evaluation of the advantage of joint modeling, as opposed to separate modeling through a comparative study of the coefficients in both model estimates from separately modeling and joint modeling. The last section discusses the results and concludes the paper.

## Materials and methods

Joint modeling is not a common approach, but it has recently garnered the attention of applied statisticians and data analysts [8–12]. Previous works on the joint modeling of correlated outcomes have largely focused on continuous outcomes or a mixture of continuous and discrete outcomes [5–10]. For example, Dunson [10] proposed a Bayesian approach for joint modeling binary and continuous subunit-specific outcomes and illustrated this approach with a developmental toxicity data example. Liang et al. [8] proposed the joint modeling and analysis of longitudinal data with informative observation times via latent variables. Additionally, Song et al. [5] investigated joint models for a time-to-event and a longitudinal response through a likelihood-based approach that requires only the assumption that the random effects have a smooth density. In light of these other studies, joint modeling is not a novel approach in statistics; however, reporting joint modeling of two correlated binary outcomes is somewhat rare. One exception to this rarity is the study of Ghebremichael [9], which identified risk factors for two binary outcomes, HSV-2 and HIV-1 infections, using a joint response model that also accommodated the interdependence between the two infections. Ghebremichael justified the advantage of a joint model using a simulation study, which showed that estimates from the joint model, as opposed to those from individual models, revealed true parameters [9]. We followed Ghebremichael [9] in applying a similar method. In addition, we accounted for the possible intra-class correlation that one typically sees in hierarchical data. In particular, we modeled two correlated responses: HIV and CUC. Each response was taken from the same subject, thus causing correlation. We further accounted for the inherent hierarchical structure because subjects are nested within enumeration areas (EAs) and can be correlated. Our method is built on joint modeling of two binary responses with a conditional distribution of underlying latent normal variables [11–14].

### Data

We used data obtained from the 2011 Bangladesh Demographic and Health Survey (BDHS). It is the sixth national-level demographic and health survey on maternal and child health in Bangladesh [1]. There are seven administrative divisions in Bangladesh: Barisal, Chittagong, Dhaka, Khulna, Rajshahi, Rangpur, and Sylhet. The survey data delineated a national representation of the entire population living in non-institutional dwelling units in Bangladesh [1]. The primary sampling unit consisted of enumeration areas (EAs). Each EA had an average of about 100 households and there were about 600 EAs. These EAs were selected with the probability proportional to size. There were 207 clusters in urban areas and 393 in rural areas. The survey data consisted of 16,186 ever-married women, ages 12 to 49, on a hierarchical structure.

The two binary responses were whether the woman a) used contraceptives or b) used a contraceptive. The covariates related to demographic status included current age, age at first marriage, the number of living children, education, religion, and place of residence. The covariates also included the living condition of the household, such as whether the household had a radio (Radio) and television (Television), as well as the family wealth index (Wealth Index). Table 1 provides the descriptive statistics of these covariates. Notably, some covariates such as education and wealth index were measured on an ordinal scale. Furthermore, there were four educational categories: no education, primary school, secondary school, and higher education and above. For each household, there was an index number to measure family income ranging from poorest, poorer, middle, richer, and richest.

**Table 1:** Summary statistics of the sample.

Variable	Code	Level	Frequency	Percent
CUC	0	no	6507	40.21
	1	yes	9677	59.79
HIV	0	no	4772	29.5
	1	yes	11412	71.5
Radio	0	no	14743	91.08
	1	yes	1443	8.92
TV	0	no	8790	54.31
	1	yes	7396	45.69
Education	0	no education	4416	27.29
	1	primary	4903	30.29
	2	secondary	5604	34.62
	3	higher	1263	7.8
Religion	1	Islam	14323	88.5
	0	other	1861	11.5
Place of Residence	1	small city	1840	11.37
	2	town	3835	23.7
	3	countryside	10509	64.93
Wealth Index	1	poorest	2865	17.7
	2	poorer	3012	18.61
	3	middle	3093	19.11
	4	richer	3403	21.03
	5	richest	3811	23.55
Divisions		Barisal	1852	11.44
		Chittagong	2579	15.94
		Dhaka	2807	17.34
		Khulna	2413	14.91
		Rajshahi	2376	14.68
		Rangpur	2289	14.14
		Sylhet	1868	11.54
	**Mean**	**Std. Dev**.	**Min**	**Max**
Age	31.42	9.19	13	49
Num of Children	2.4	1.59	0	10
Age at Marriage	15.65	2.94	10	48

The average age of the women in the survey was 31 years old, with the mean age of the first marriage at 15 years old. On average, each woman had between two and three children, and about 60% of the women used contraceptives. Notably, religion played an important part in the respondents’ beliefs, with 90% of the sample being Muslim. Geographically, approximately 65% of respondents lived in rural areas, as compared to 35% who lived in urban areas. Specifically, education was lacking among Bangladesh women because only 8% had received higher education, as compared to 27% who had never received any education. Regarding living conditions, approximately 46% of households had a television at home, but only 9% of them had a radio. Based on the Wealth Index, 17.70% of the population fell into the poorest category, 18.61% were classified as poorer, 19.11% were defined as middle class, 21.03% were classified as richer, and 23.55% were defined as richest.

### Statistical analysis

Let *Y*_*ij*_ be a binary random variable and take the values of one or zero that denotes the *i*^*th*^ outcome (i = 1, 2) of the *i*^*th*^ (j = 1, 2, …, n = 16,186) subject, with i = 1 for contraceptive use and i = 2 for knowledge of HIV. The two binary outcomes give rise to a bivariate binary response vector $Y=(\begin{array}{c} Y_{1j}\\ Y_{2j} \end{array})$ with a corresponding data matrix of covariates (*X*_1*j*_, *X*_2*j*_) where *X*_1*j*_ = (1, *X*_*ij*1_, … *X*_*ijp*_) and corresponding vectors of regression coefficients (*β*_1_, *β*_2_). *Z*_1_ is the matrix of random effects with corresponding coefficients *γ*. Covariates included number of children (Num of Children), age at first marriage (Age at Marriage), current age (Age), radio (Radio), television (Television), educational levels (Education), religious belief (Religion) and family wealth index (Wealth Index). Each woman has two outcomes (HIV or CUC), and since condom use and other HIV education overlap, these outcomes are correlated.

We fitted joint hierarchical logistic regression models with shared random effects that accounted for the correlation due to clusters and divisions. The shared random intercept accounted for the correlation between the two responses of the same individual. This random intercept captures the unobserved factors specific to each individual that may influence both contraceptive use and HIV knowledge [15, 16]. Thus, considering the additional random intercept we presented the joint model as:

$$\begin{array}{c} \begin{array}{c} logit\left[P_{i}|X_{ij},\beta_{i},u_{j}\right]=I_{1j}(\beta_{0}+\beta_{1}X_{1jk}+\ldots \beta_{p}X_{pjk}+\gamma 0_{clu}+\gamma 1_{clu}Z_{jk}+\\ \gamma 0_{div}+\gamma 1_{div}Z_{j^{\prime }k^{\prime }})+I_{2j}(\beta_{0}+\beta_{1}X_{1jk}+\ldots \beta_{p}X_{pjk}+\\ \gamma 0_{clu}+\gamma 1_{clu}Z_{jk}+\gamma 0_{div}+\gamma 1_{div}Z_{j^{\prime }k^{\prime }}) \end{array}, \end{array}$$ (1)

where *I*_*ij*_ is the indicator function for outcome i, *u*_*j*_ are the random intercepts assumed to be normally distributed with mean zero and variance $\sigma_{u}^{2}$ and *P*_*i*_ represents *P*_*CUC*_ when i = 1 and *P*_*HIV*_ when i = 2. These estimators are consistent and asymptotically normally distributed. We assume that there are unobserved latent factors that provide random effects between HIV and CUC by the same subject. As such, the two outcomes are correlated within each subject. We account for this through a shared random intercept: this random intercept is meant to capture the unobserved factor within each subject and may have an influence on the responses. Conditioned on the shared random intercept, the joint responses of a woman are assumed independent. Through conditional independence assumption, one can obtain the likelihood function of the joint responses [9]. Following Ghebremichael [9], We used a maximum likelihood approach to obtain estimates of the regression parameters and the covariance parameters. As the integrals do not have closed-form, we used an approximation based on Gaussian quadrature, as found to be consistent and asymptotically normally distributed by [9].

## Results and conclusion

As discussed, we fitted two sets of models: separate models and a joint model. In the separate models, we used a hierarchical logistic regression. To validate that there were indeed variation within individual, cluster (EA), and division, we first performed a Hosmer and Lemeshow test within each level and our test results suggested a hierarchical structure. The over-dispersion of variance due to the Hosmer and Lemeshow test value is χ^2^ = 87.859 (p-value < 0.001), indicating that multi-level logistic model is needed. Therefore, we fitted a three-level hierarchical logistic regression with random intercepts and random slopes to address these issues of intraclass correlation. We found that the variance at division (var(division) $\sigma_{div}^{2}=0$ with p < 0.0001) and the variance at cluster (var(cluster(division))$\sigma_{clu(div)}^{2}=0$ with p = 0.0125) are both significantly different from zero. Therefore, our model assumed that the random effects *γ*_0_*clu*__ and *γ*_0_*div*__ each represents a random sample from a normal distribution with variance of 0.050 for clusters and 0.204 for divisions, respectively. We presented results in Table 2 with the odds ratio of the parameter estimates.

**Table 2:** Results for three-level hierarchical models and joint model.

	Contraceptive Use				HIV Knowledge			
Variable	Joint Model		Three-Level		Joint Model		Three-Level	
	*Odds Ratio*	*p-value*	*Odds Ratio*	*p-value*	*Odds Ratio*	*p-value*	*Odds Ratio*	*p-value*
Intercept	1.60	<0.001	2.53	<0.001	1.73	<0.001	0.33	<0.001
Age	0.99	<0.001	0.94	<0.001	1.00	0.002	0.99	0.004
Age at marriage	1.01	<0.001	1.03	<0.001	1.00	0.016	1.03	0.002
Children	1.10	<0.001	1.55	<0.001	0.98	<0.001	0.92	<0.001
Radio	1.00	0.825	1.03	0.628	1.03	0.005	1.26	0.003
Television	1.03	0.005	1.14	0.01	1.10	<0.001	1.90	<0.001
Education	1.03	0.045	1.12	<0.001	1.16	<0.001	2.68	<0.001
Religion	0.91	<0.001	0.66	<0.001	1.02	0.016	1.10	0.206
Urban	1.05	<0.001	1.28	<0.001	1.04	<0.001	1.73	<0.001
Wealth index	0.99	0.142	0.99	0.621	1.05	<0.001	1.26	<0.001

We found that social demographic factors such as age, age at marriage, number of children, and education are statistically significant in influencing contraceptive use. More specifically, we observed that older women were less likely to use contraceptives, women who got married late are more likely to use contraceptives, and contraceptive methods were more likely to be employed as the number of children increased. Furthermore, as a woman’s education level increased, she was more likely to use contraceptive methods. Regarding living conditions, we found that having a television increased the likelihood of using contraceptive; however, having a radio did not. We did not find household wealth to be a significant factor for CUC, even though education and living conditions can be related to wealth. This may be the result of the fact that contraceptive use was a common practice that applied to the wealthy and the poor together; therefore, the financial status did not play an important part. Islam was the major belief in Bangladesh. As the literature shows, being Muslim reduces one’s likelihood of using contraceptives [17], a congruence that appeared in our results.

We found similar driving factors for HIV knowledge as those obtained for CUC. Older women and women who got married early are less likely to have knowledge about HIV. The more children one had, the more likely she had knowledge of HIV. Education played a paramount role in the likelihood of HIV knowledge. Urban women were more likely to have knowledge of HIV than rural women were. In addition, women who had radio and TV at home were more likely to have HIV knowledge. Unlike CUC, HIV was positively driven by the household wealth: wealthier families were more likely to have HIV knowledge than less wealthy families. These findings shed light on the crucial importance of education and media on HIV prevention. Furthermore, religion did not play an important part in HIV knowledge.

The results regarding CUC from a separate hierarchical logistic regression model revealed that estimates obtained for age, age at marriage, children, TV, education, religion, and urban were found to be significant with the same incremental impact. Estimates obtained for radio and wealth index were insignificant, as in the case of the joint model. Additionally, we found that CUC had a strong divisional random effect. Rajshahi had the largest differential random effects on contraceptive methods, followed by Rangpur.

As for HIV knowledge, we found the same incremental significance for all covariates, except age at marriage, as in separate models. In the separate model, women who got married late were less likely to have HIV knowledge. However, the estimate from the joint model told the opposing story: women who got married late were more likely to have HIV knowledge. Estimates from the joint model made intuitive sense because women in Bangladesh tend to marry young, which consequently deprived them the opportunity for HIV education. Moreover, women from less wealthy families were less likely to have TV or radio, which limits their exposure to media about HIV. These women were the ones who got married early. The distinction, which depends on the correlation through the shared random effects, between these two approaches is crucial: when we failed to address the correlation between CUC and HIV, it led to an overestimation of the relation between the covariates and the outcomes (Table 2).

Ignoring the random effects of division and EA would underestimate the effect of education. For example, if we had modeled CUC without any adjustment for divisional and EA random effects, one may conclude that education will likely increase CUC by likelihood for 0.067. However, using a model that accounted for the variation in different geographic divisions and sampling units (EA), the magnitude of education on CUC increased to 0.128, which translates to an increase in likelihood. More importantly, after taking into account the correlation between HIV knowledge and contraceptive use, the importance of education decreases to 0.033. This means that the HIV prevention program has a spillover effect on CUC, and what seems to be an impact from education can be partially contributed to one’s exposure to HIV knowledge. As for modeling the HIV knowledge, we also observed estimates that joint models revealed as smaller values in magnitude compared to separate models and standard models. These findings substantiated the need for jointly modeling two correlated outcomes. By so doing, we accounted for the variation between two outcomes and deflated the otherwise overestimated parameters from separate models.

## Discussion

Family planning policy in Bangladesh has made great strides in the past four decades through the increased contraceptive use. Still, while HIV prevalence is low in Bangladesh, it presents a steadily increasing trend. The emergence of a generalized HIV epidemic would be a disaster that poverty-stricken Bangladesh could not afford [3]. Interested in addressing both events more efficiently, we fitted a series of statistical models using the BDHS2011 data. Consistent with previous research, we found that age at marriage was negatively associated with contraceptive use but positively associated with the knowledge of HIV. Women who were older, had a higher educational level, and had more children are more exposed to contraceptive use and HIV knowledge. Religious beliefs decreased the likelihood of using contraceptive methods but increased the knowledge of HIV. We found radio ownership, as an agent of media, to be significant in influencing HIV knowledge but not contraceptive use. Moreover, financial status had no significant impact on contraceptive use, unlike in the case of HIV knowledge. These two findings showed that contraceptive use was relatively well acknowledged and practiced regardless of the household income and social status. On the other hand, HIV knowledge was still novel to some people. Therefore, education, social media, and social status often correlated with education and were important in promoting HIV knowledge.

The prevalence rates of contraceptive use and knowledge about HIV in Bangladesh were 90% and 70%, respectively, indicating a likelihood of overlap between these two events. Thus, we used a joint response model that accommodated the interdependence between contraceptive use and knowledge about AIDS. Our findings contributed to the understanding that joint models deflate parameters that are otherwise overestimated in separate models. We found that the strength of the relationship was higher in absolute values from separate models, causing false interpretations of the attributes in the magnitude. To correctly translate the importance of certain covariates, a researcher should take into account the correlated outcomes caused by common covariates. In this case, after accounting for the correlation between CUC and HIV knowledge in simultaneous estimation, we found strengths of the relationship to be smaller than those in separate models did.

The implication regarding public health is straightforward: when we have two social programs that aim to have a similar impact, a positive spillover effect from one to the other exists. By treating them independently, we may put too much emphasis on the covariates. For example, treating the two events separately, we may recommend a budget increase to put girls in school. Alternatively, if we included the interdependency of the programs, we will realize that the importance of education on CUC is actually compensated by the education on HIV.

Many developing countries have substantial geographic variations in contraceptive use, although the factors driving these variations are not always understood. Previous studies showed that variations in contraceptive use typically remained after accounting for individual and household factors. We found that both CUC and HIV knowledge varied within enumeration areas and across divisions, causing cluster effects to be significant. This indicated that policies should be tailored to cater to specific geo-social differences. Although we did not analyze the specific driving factors that contribute to geographical variations, our joint model showed that they were the same contextual factors that drive both CUC and HIV knowledge. Future research should look into community-level cultural beliefs, the availability of health services, the physical characteristics of the area, macroeconomic factors, and the presence of transport routes in order to explain the variations in geographical difference for CUC and HIV knowledge.
